# Quantitative In Vivo Genetic Analysis Reveals Novel Genetic Determinants of Tumor Initiation, Overall Growth, and Exceptional Growth in KRAS-Driven Lung Cancer

**DOI:** 10.1055/s-0041-1736238

**Published:** 2021-10-05

**Authors:** Ji Ruan

**Affiliations:** 1 State Key Laboratory of Oncology in South China, Collaborative Innovation Center for Cancer Medicine, Sun Yat-sen University Cancer Center, Guangzhou, People's Republic of China

Cancer is one of the major burdens of disease and leading causes of death in the world. 1 With more than 1.4 billion populations, China contributes more than 25% of the global cancer burden. 2 3 Therefore, there is an urgent need for better understanding the molecular mechanisms of cancer initiation and development to identify novel targets for cancer therapy and prevention. Cancer initiation and development is a multistep process mainly driven by the accumulation of genetic/epigenetic alterations in multiple genes, including oncogenes and tumor suppressor genes. 4 In the past few decades, genome sequencing has been the major method to identify driver genes in cancer, especially tumor suppressor genes. 5 6 7 However, genome sequencing cannot determine at which stage these genes play a driving role in cancer development. 8 In vivo functional genomic approaches can address this deficiency in genome sequencing. 9

As we have known, abnormal tumor suppressor genes are involved in the regulation of many different cellular processes and signaling pathways of cancer cells. Determining their functions in each step of cancer initiation and development can effectively distinguish them from passenger genes and further determine different processes and signaling pathways that limit tumorigenesis in the entire disease process. 10 Therefore, in vivo functional genomics approaches are currently essential for exploring the molecular mechanisms of cancer development, interpreting cancer genome sequencing g data, developing new anti-tumor drugs, and guiding cancer precision medicine. 11 As primary tumors occurred in animal models are initiate and grow in the autochthonous environment entirely, they are uniquely tractable systems to facilitate the discovery of the cancer-related gene functions. 12 Recently, the strategy of applying CRISPR/Cas9 genome editing to genetically-modified animal models of human cancer has greatly facilitated the rapid analysis of cancer-related gene functions in vivo. 13 Furthermore, since the impact of many engineered genome changes on tumor growth in vivo can be quantified in many ways, the combination of CRISPR/Cas9-based genome editing with tumor barcoding coupled with high-throughput barcode sequencing (Tuba-seq) has greatly improved the detection scale and accuracy of the in vivo functional genomics approaches. 14 In a study recently published in *Cancer Discovery* , titled “A functional taxonomy of tumor suppression in oncogenic KRAS-driven lung cancer,” Cai et al 15 uncovered a complex taxonomy of tumor suppression across the life history of KRAS-driven lung cancer by integrating multiple key advances of lung cancer in Tuba-seq pipeline and quantifying the roles of different tumor suppressors in controlling lung cancer development, growth, and changing phenotypes over a period of time.

In this study, the authors selected 48 well-established and putative tumor suppressor genes based on their mutational frequency in lung cancer to characterize the landscape of tumor suppression function in a multiplexed KRAS-driven lung cancer model. After inactivation of each selected gene by CRISPR/Cas9 technique, they quantified the tumor size profiles of KRAS-driven lung cancer model by using Tuba-seq to determine the impact of inactivating each putative tumor suppressor gene on tumorigenesis in vivo. To further investigate which aspects of tumorigenesis are regulated by candidate tumor suppressor genes, the authors calculated several statistics that can reflect the restriction of overall tumor growth, tumor initiation, and the appearance of abnormally large tumors. They identified many novel tumor suppressor genes in KRAS-driven lung cancer and revealed commonality among lung cancer subtypes. Most notable among these genes is *STAG2* , which encodes a component of cohesin complex. Inactivation of STAG2 increased overall tumor growth in KRAS-driven lung cancer model to a comparable extent as inactivation of those well-studied tumor suppressor genes. Next, tumor suppressor gene function at multiple time points after tumor initiation was assessed to gain the temporal resolution of tumor suppressor effects in lung cancer. This method captured other undiscovered aspects of tumor suppressor gene function. Furthermore, to validate the effect of tumor suppressor gene inactivation on tumor initiation, the authors analyzed the effect of each genotype in mouse lung cancer model initiated with Lenti- *sg85* /Cre pool. They found that there is a close connection between the cellular alterations that enable epithelial cells to break through the limitations of hyperplastic growth and the greater fitness in the resulting tumors. By using Tuba-seq data to quantify the impact of candidate tumor suppressor gene inactivation on the generation of rare but very large tumors, the authors found that the role of tumor suppressor genes in preventing the generation of very large tumors can be independent of their role in the regulation of tumors during tumor evolution.

Genomic diversity is a huge obstacle to identifying driver genes and deciphering the role of driver genes in cancer development. As the sample size of cancer genome sequencing is limited, variation in genomic characteristics seriously affects the accuracy of computational predictions of tumor suppressor functions based on mutation data. 8 In addition, mutation frequencies alone cannot accurately distinguish driver genes from passenger genes in cancer, and even cannot be used for functional mechanism prediction of driver genes. Indeed, some tumor suppressor genes with rare mutations can cause serious consequences when they are inactivated. 8 Therefore, studying the function of human tumor suppressor genes in animal models is an important complement to computational investigation, even though species specificity will have a certain impact on this research strategy.

Cancer development is affected by various aspects of the in vivo environment. By applying Tuba-seq which can carry higher throughput and has greater sensitivity and accuracy, the present study quantified the effects of inactivating a series of tumor suppressor candidate genes in a KRAS-driven lung cancer mouse model. Parallel analysis of different genotypes not only identified new functional tumor suppressor genes, but also revealed their multiple modes of action. The results of parallel analysis showed that tumor suppression in KRAS-driven lung cancer model was very complex. For example, some genes inhibit the growth of tumors as a whole, while others restrict the emergence of only a small number of abnormally fast-growing tumors. In addition, some genes affect only a single feature of cancer development, while some genes affect multiple aspects of tumor evolution. The relative importance of these genes can be constantly regulated and dynamically changed during the process of tumorigenesis. These findings provided new information for tumor suppression landscape.

The results of this study are similar to previous single-gene studies using the same animal models, 13 14 but the single-gene approaches cannot reveal tumor suppression modes, and it is impossible to make direct comparisons across many genotypes. However, the multiplexed design and computational platform of this study uniquely achieve the deconvolution of different aspects of tumor suppression. Moreover, this study has discovered many new tumor suppressor candidates that have a decisive effect on tumor growth. Identifying these tumor suppressor genes, especially those that are not frequently mutated in lung cancer, can highlight key signaling pathways and cellular processes that are critical in cancer.

Analyzing the mutual exclusivity in human data is a key approach used to reveal the context dependency of tumor suppressor function. 16 Most of the genes that are mutually exclusive with KRAS mutations found in this study are important suppressors of KRAS-driven lung cancer that have been reported in previous studies, such as PTEN and NF1. The statistical trend toward mutual exclusivity should be interpreted with caution and should not be misinterpreted as the lack of tumor suppressive effects of these genes in KRAS-driven lung cancer.

Overall, this study provides a comprehensive map of in vivo functions of tumor suppressor genes for cancer. In the future, we can use this method to further analyze cancers with different genetic and environmental backgrounds to further clarify the mode and context dependence of tumor suppressor gene effects.
